# Immobilization of the Enzyme Glucose Oxidase on Both Bulk and Porous SiO_2_ Surfaces

**DOI:** 10.3390/s8095637

**Published:** 2008-09-15

**Authors:** Sebania Libertino, Venera Aiello, Antonino Scandurra, Marcella Renis, Fulvia Sinatra

**Affiliations:** 1 CNR – IMM Stradale Primosole 50 Catania, Italy; 2 Università degli Studi di Catania, Dipartimento di Chimica Biologica, Chimica Medica e Biologia Molecolare, Catania, Italy; E-Mails: vera.aiello@imm.cnr.it (V. A.); renis@unict.it (M. R.); 3 Università degli Studi di Catania, Dipartimento di Scienze Biomediche, Catania, Italy;E-mail: sinatra@unict.it (F. S.); 4 Laboratorio Superfici e Interfasi (SUPERLAB), Consorzio Catania Ricerche, Catania, Italy;E-mail: ascandura@unict.it (A. S.)

**Keywords:** Glucose oxidase, oxide activation, glutaraldehyde, enzymatic activity, spectrophotometric assay, biosensor

## Abstract

Silicon dioxide surfaces, both bulk and porous, were used to anchor the enzyme glucose oxidase. The immobilization protocol was optimized and the samples characterized using X-ray Photoelectron Spectroscopy, Energy Dispersive X-rays coupled to scanning electron microscopy and enzymatic activity measurements. We show that a uniform layer was obtained by activating the oxide before immobilization. X-ray Photoelectron Spectroscopy measurements carried out on bulk oxide showed that the silicon substrate signal was fully screened after the enzyme deposition showing the absence of uncovered surface regions. The enzyme presence was detected monitoring both the C 1*s* and N 1*s* signals. Finally, enzymatic activity measurements confirmed that the glucose oxidase activity was preserved after immobilization and maintained after three months of shelf life if the sample was properly stored. The importance of using porous silicon oxide to maximize the surface area was also evidenced.

## 1. Introduction

The increasing importance of biosensors in everyday life is the driving force behind a merging of the microelectronic and biomedical communities. The common effort is the production of devices ready for mass production that will perform accurate analyses. Among microelectronic materials, silicon (Si) has the most mature and low cost technology, hence, several research groups are approaching Si compatible technology as innovative platform for biosensors. Moreover, Si based matrixes have been proved to be a very useful support for the immobilization of enzymes thanks to their capability of retaining biological activity. To develop a useful device, particular care must be used for biological molecule immobilization on Si-based surfaces. There is a body of data in the literature [1, 2] regarding immobilization mechanisms of biological molecules on solid surfaces. The most used approach is the formation of covalent bonds with the solid surface [1-7], often using bifunctional reagents to bridge the biological molecule and the functionalized sample surface.

Si-based biosensors, as well as conventional microelectronic devices, must be fully characterized using standard microelectronics techniques allowing biological molecule monitoring. In this way, the new technology costs are contained, since no new equipment is needed. Among the different techniques, X-ray Photoelectron Spectroscopy (XPS) and Energy Dispersive X-rays (EDX) coupled to Scanning Electron Microscopy (SEM) are very intriguing. The first one provides information on chemical bonds and molecular composition of the material surfaces, combined with a high surface specificity, while the second one, when operated on cross-sections, allows one the investigation of thick layers of material without, if coupled to SEM, complex sample preparation.

The above mentioned techniques were used to study the immobilization of glucose oxidase (GOx) enzyme from *Aspergillus niger*, on an SiO_2_ surface. GOx is a dimeric globular protein having overall dimensions of 6.0×5.2×7.7 nm^3^. It catalyzes the oxidation of β-d-glucose to δ-gluconolactone by a reaction that can be summarized in two steps: i) glucose oxidation with the enzyme reduction, ii) re-oxidation of the enzyme with consumption of molecular oxide (O_2_) and production of hydrogen peroxide (H_2_O_2_) [8]. For this reason GOx is commonly used to monitor the glucose concentration in the blood; hence, a GOx based micro-biosensor [9] would have immediate applications in monitoring diabetes [10].

In order to visualize the enzyme with XPS, it was labelled with gold nanoparticles having a diameter of 1.4 nm. Au is regularly used to label the amino groups of biological molecules in solution when transmission electron microscopy measurements are performed [11]. GOx molecules contain two disulphide bonds and two accessible thiol groups [12]. Since GOx amino groups were used to immobilize the protein on the solid surface, the free sulfydryl groups were labelled with functionalized gold nanoparticles [13]. Gold labelling in this study had a twofold purpose: to provide conclusive proof of the presence of the enzyme and as internal reference for XPS measurements. To verify the preservation of enzyme activity after immobilization, a spectrophotometric technique was used. It was based on the use of peroxidase to monitor hydrogen peroxide production.

The aims of this work were the definition of an optimized immobilization protocol that would allow the maximum surface coverage without any loss in the GOx enzyme performances and the characterization of the deposited organic layer using standard microelectronic techniques.

## 2. Results and Discussion

**Figure 1.**
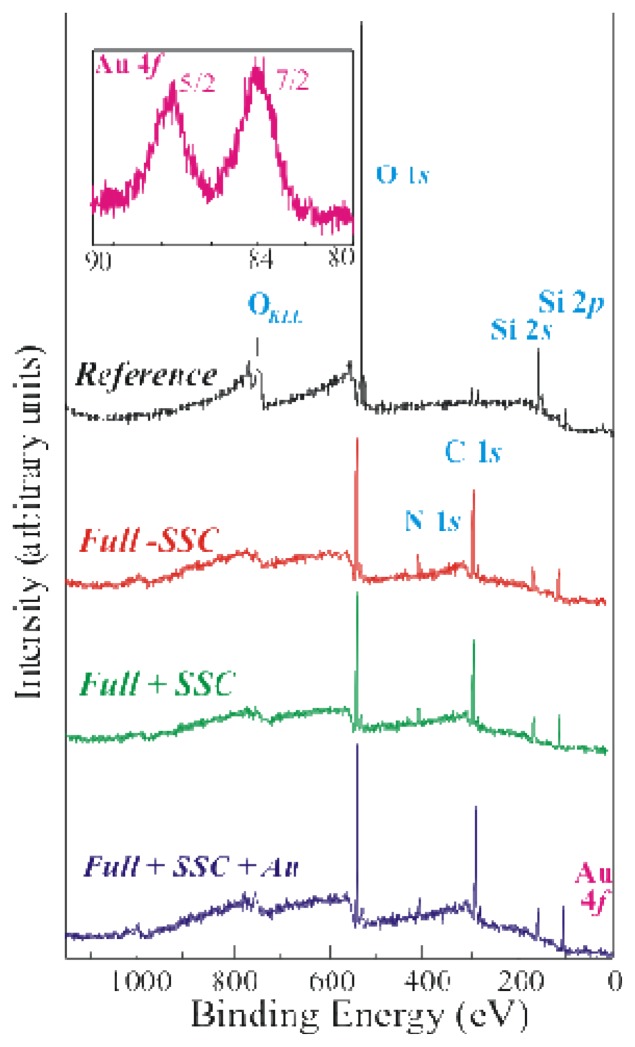
XPS survey spectra of reference (black) and three fully processed samples: processed without SSC (red, Full-SSC), processed with SSC (green, Full+SSC), processed with SSC and GOx labelled with Au nanoparticles (blue, Full+SSC+Au). In the inset, a zoom between 80eV and 93eV showing the Au 4*f* shell signal of the last mentioned sample. No signal was observed in the other samples.

The most promising immobilization protocol reported in literature (see ref. [2]) was tested on bulk SiO_2_ samples, mainly using XPS measurements. The results were compared with those obtained from a sample where a modified protocol was applied. The main difference with respect to the fist protocol was the addition of an oxide activation step at the beginning of the processing (see the experimental section for details). The XPS survey spectra of the reference (top spectrum, in black) and three fully processed samples (differently prepared) are shown in Figure 1.

The second spectrum from the top was acquired from a sample obtained after the three step procedure reported in the literature [2] (Full without SSC, Full-SSC, in red), while the other two spectra were obtained from samples where the modified protocol was applied (full +SSC, in green and in blue). GOx was labelled with Au nanoparticles before immobilization during the last sample preparation (Full+SSC+Au, in blue).

The reference sample exhibited the XPS Si peaks centred at about 155 eV (Si 2*s*) and 104 eV (Si 2*p*), a very small C peak (due to adventitious contamination) centred at about 285 eV and the O 1*s* XPS signal centred at a binding energy of about 533eV. All the fully processed samples show the same signature already observed for the reference sample, even if their relative concentrations are quite different. As an example the C 1*s* peak was well visible for all these samples, thanks to the presence of organic material. Moreover the N 1*s* peak at about 400eV was detected in these spectra to demonstrate the enzyme presence in the fully processed samples [14]. Finally, the Au 4*f* peak was detected in the Full+SSC+Au sample. The expanded spectral region of Au *4f* for this sample is shown in the inset of Figure 1. The doublet Au*4f*_7/2_ and Au*4f*_5/2_ (used as reference) exhibited binding energies of 84.0 and 87.7eV, respectively. Au presence provided a conclusive proof of GOx immobilization on the sample. It should be mentioned that the Au peaks observed in XPS are a direct experimental evidence of the GOx presence on the sample. Literature results provide only indirect evidences of GOx immobilization, obtained using its enzymatic activity (as an example see refs. [4, 5]).

The three fully processed samples, prepared using three different methods (Full+SSC, Full-SSC and Full+SSC+Au) allowed us to answer many open questions on the goodness of our protocol. First of all, the comparison with a protocol widely used in literature allowed us to directly measure any improvement in the sample preparation due to the introduction of a further step (SSC treatment). Moreover, gold labelling provided an experimental direct proof of the GOx presence, not found, to our knowledge, in literature.

**Figure 2.**
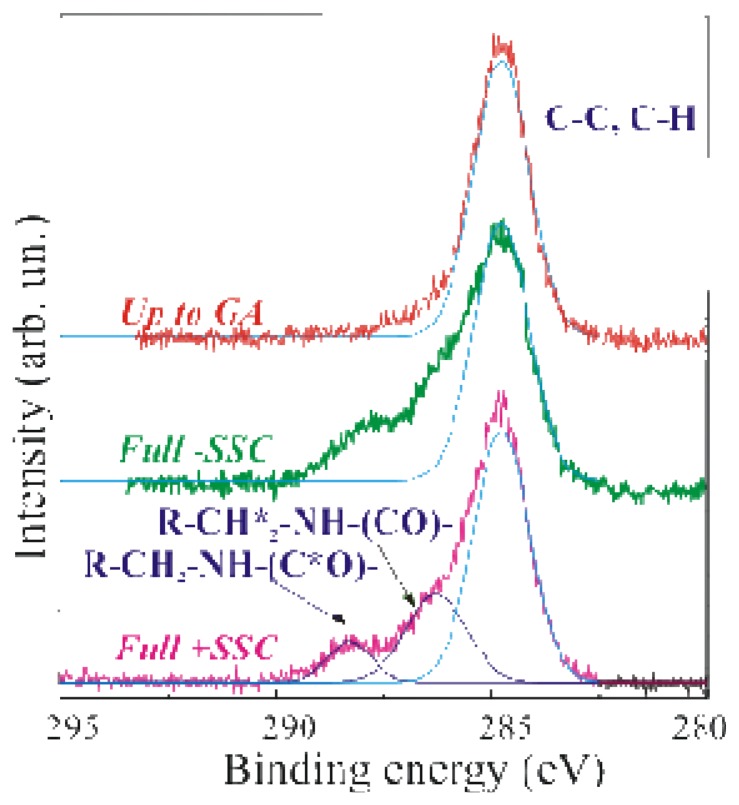
High acquisition mode XPS C1*s* spectral regions of: up-to-GA (red line), fully processed without SSC (green line, Full-SSC) and fully processed with SSC (magenta line, Full+SSC) samples. The light and dark blue lines are the simulated peaks.

A further confirmation of the GOx presence was easily obtained by the inspection of the C 1*s* peak reported in Figure 2, where the XPS spectra of a sample stopped after the GA immobilization step (SSC+APTES+GA, labelled up-to-GA, red line), the Full-SSC (green line) and the Fully processed (Full+SSC, magenta line) samples are compared in the binding energy range 295–280eV.

The C 1*s* peak, centred at 284.8–285eV, is due to C-C and C-H bonds. The light blue line superimposed to the experimental spectrum was a simulation of the C-C and C-H XPS peak. The up-to-GA sample showed only this peak, and an additional weak shoulder at about 287eV attributed to the R-CHO groups of GA. It should be mentioned that the GA is a linear molecule (CHO-(CH_2_)_3_-CHO) with one aldehydic group (CHO) at each end. The other two samples showed at least other two components at 286.3–286.5eV, assigned to R-CH_2_*-NH-(CO)-, and at 288.3eV, assigned to R-CH_2_-NH-(C*O)-chemical groups respectively. The dark blue lines in Figure 2 are the simulated peaks superimposed to the experimental data to allow one an easier identification of the different peaks. Due to the sensitivity of the XPS technique, phosphorous and the other functions of the FAD groups were not detected.

The comparison of the two different preparation methods (Full-SSC and Full+SSC) allowed us to conclude that the C peak inspection does not allow to observe differences between our immobilization protocol and the former (Full-SSC). A more interesting data to compare the two immobilization methods is provided by the inspection of the Si related peaks.

**Figure 3.**
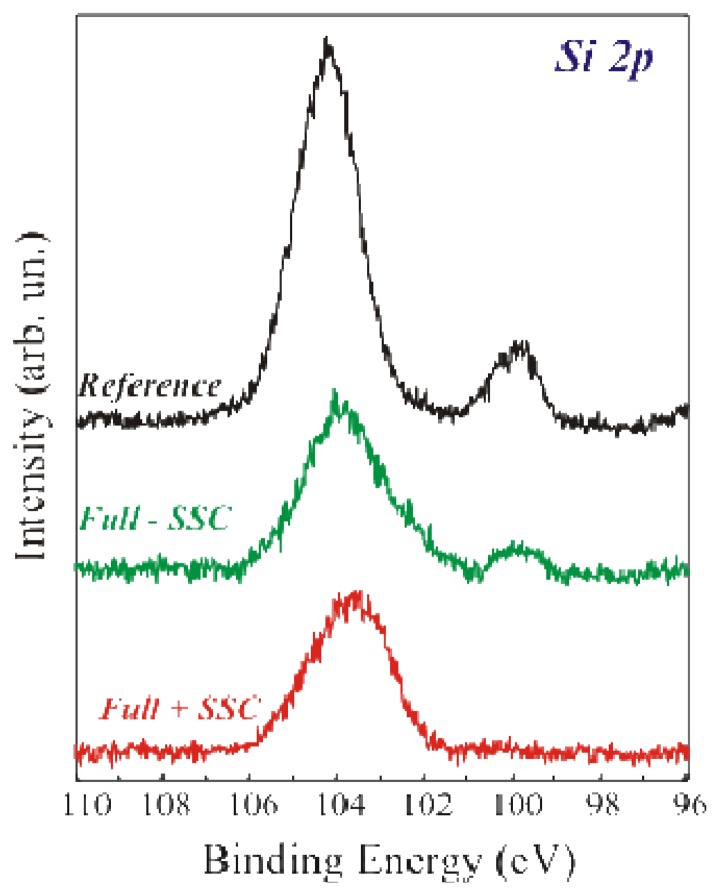
High acquisition mode XPS Si2*p* spectral region of the reference (black line), fully processed without SSC (green line, Full-SSC) and fully process with SSC (red line) samples.

The Si 2*p* spectra of the above mentioned samples are shown in Figure 3. The reference sample (in black) exhibited two components having binding energies of 99.7 eV and 104 eV, assigned to Si° and SiO_2_ respectively [15, 16]. The SiO_2_ component is centred at 104 eV instead of 103.4 eV, as expected, due to differential charging between the SiO_2_ layer and the Si substrate [17]. The Full+SSC sample (bottom spectrum) does not exhibit the Si° component, while the Full-SSC sample (centre spectrum) still exhibits the Si° component.

Recent literature data confirm that if the SiO_2_ layer is thin (6.5 nm in our case), it is possible to infer information on the uniformity of the biological layer [13, 14]. If the coverage was uniform, after GA immobilization, there should be a film fully shielding the Si° signal from the Si under the oxide. Two conditions must be fulfilled to have a complete substrate shield. The first one is a film thickness above the photoelectron penetration depth. The second is the uniformity of APTES+GA and, eventually, +GOx layer. The real shielding was confirmed by our measurements. In fact, the fully processed sample Si 2*p* spectrum showed only SiO_2_ component. The intensity of the Si substrate was attenuated by the two added layers (SiO_2_+organic) [14]. On the other hand, if the immobilization procedure did not produce a uniform film, the Si° signal was still visible after the full immobilization protocol, despite of the fact that the enzyme was correctly immobilized, as demonstrated by the Full-SSC spectrum. The results confirmed that the immobilization procedure without oxide activation was not the best achievable on our SiO_2_ surfaces. This conclusion was possible only thanks to the direct comparison of the two immobilization methods on the same kind of sample (oxide thickness and SiO_2_ preparation method).

The great improvement in film uniformity measured on the samples that underwent Full+SSC protocol can be explained considering the SiO_2_ growing procedure. In fact, the oxide layer was grown in a dry environment (see experimental). Recent studies [18] have demonstrated that silicon oxides grown in a dry environment have a density of Si–OH surface endings lower than wet grown oxides. As a result, they have a lower reactivity against silanization, leading, in our case, to a lower enzyme uniformity. The oxide activation we introduced as the first step allowed us to increase the number of available sites for enzyme bonding and to obtain a final uniform deposition. The enzymatic activity measurements confirmed these results (see after).

**Figure 4.**
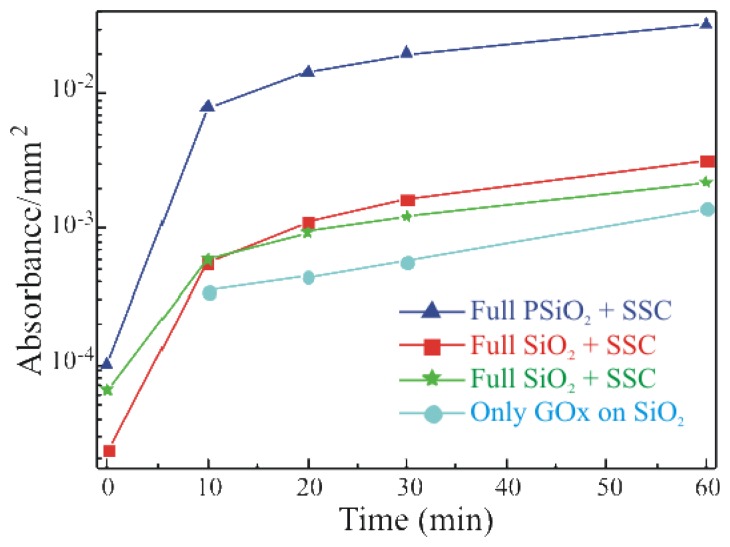
Glucose oxidase activity as a function of time for: bulk SiO_2_ sample fully processed without SSC (green stars, Full-SSC), bulk SiO_2_ sample fully processed with SSC (red squares, Full+SSC), bulk SiO_2_ only GOx (light blue circles), porous SiO_2_ sample fully processed with SSC (dark blue triangles).

To study the activity of the immobilized enzyme, a simple, spectrophotometric assay was used. GOx activity was monitored on the Full+SSC sample, on the Full-SSC (former protocol) sample and on the sample that underwent only GOx deposition without previous surface functionalization (only GOx). The results are summarized in Figure 4 and compared with the activity of a porous silicon dioxide (PSiO_2_) fully processed with SSC sample. All data were normalized to the real area of the sample.

The data show an increase in the enzymatic activity when the oxide activation is carried out before silanization (Full+SSC; red squares) with respect to the Full-SSC sample (green stars). The only-GOx sample (light blue circles) exhibited an enzymatic activity lower than the one measured for both the samples that underwent chemical immobilization. The comparison of the absorbance values with those obtained from the free enzymes in solution, allowed us to estimate a concentration of active GOx on the SiO_2_ samples of about 0.002 U ml^-1^[21]. The immobilization protocol carried out on porous SiO_2_ surfaces (dark blue triangles) clearly indicated a higher enzymatic activity, more than one order of magnitude, for this sample with respect to the bulk SiO_2_ samples. It is due to the greater surface available in such material. In fact, PSiO_2_ has a porous structure, like a sponge, hence a greater surface to volume ratio. Values up to 1000 can be obtained. An SEM image of the PSiO_2_ layer is provided in the inset of Figure 6 Now, if the GOx permeates the PSiO_2_ pores a higher enzyme amount can be deposited on the same sample area with respect to a bulk SiO_2_ surface. Similar results are reported in literature [19] and provide an indirect measure of the enzyme presence within the pores.

**Figure 5.**
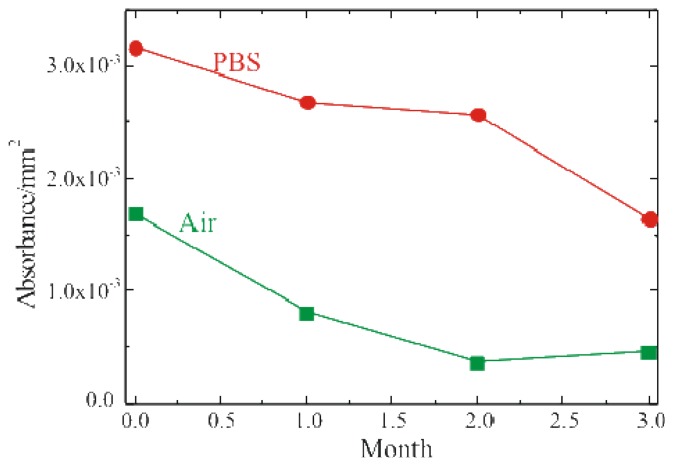
Glucose oxidase activity as a function of storage time for bulk SiO_2_ samples stored in PBS at 4°C (red dots) and in air at RT (green squares).

The enzymatic activity of the fully processed sample, stored either in buffer solution (PBS, 0.1M pH 6.5) at 4°C or in air at RT, was monitored. The results of these measurements are summarized in Figure 5 where the red circles represent the sample stored in PBS, while the green squares represent the sample stored in air. Both samples were monitored immediately after immobilization and after 1, 2 and 3 months. The absorbance values reported in figure were detected 60 min after the reaction started. The samples stored in PBS retained activity over longer periods of time. On the other hand, the samples stored in air showed an immediate decrease in the enzymatic activity, already after the first month of aging.

The presence of carbon in the full porous thickness confirmed the result already observed from the enzymatic activity measurements: the enzyme permeated the pores. This last measurement provides a direct experimental evidence of the enzyme presence in pores, while its functionality is witnessed by the spectrophotometric measurements of Figure 4.

**Figure 6.**
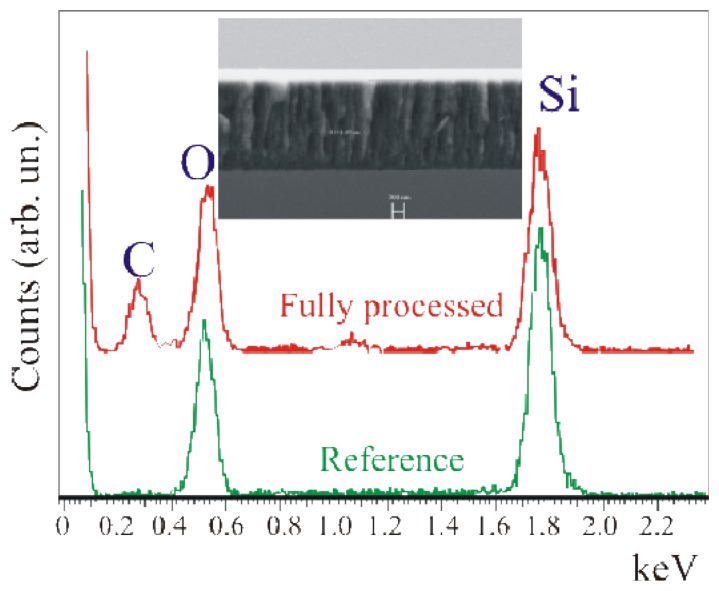
EDX measurements coupled to SEM on the cross section for the reference (green line) and the fully processed (Full+SSC, red line) samples. Both measurements were acquired close to the porous SiO_2_/Si interface. In the insert the SEM image of a porous SiO_2_ sample cross section. The marker is 300 nm.

Using SEM-EDX measurements [22] some interesting observations were made (Figure 6). Both the thickness and the porous nature of the layer are clearly visible in the insert of Figure 6, where the SEM image of the device cross-section shows a layer thickness of 2.26 μm. EDX measurements were carried out on the fully processed sample (Full + SSC) and on a reference sample. This comparison is needed to exclude any carbon contamination of the porous layer. In fact, since the PSi is obtained through electrochemical reaction in a cell containing a C-rich solution (see Experimental), the sample could absorb C (or have C bonded to the surface) after processing. Such C could diffuse in the sample during the subsequent oxidation process. As a result a C peak not necessarily related to the biological molecules could be detected. In our case, the reference did not show any C contamination (within the detection limits) and exhibited only the presence of O and Si, as observed from the K_α_ emission of the two elements at 0.51 keV and 1.73 keV, respectively. On the other hand, the sample that underwent the full immobilization protocol exhibited, in addition to the Si and O signals, a clear emission at 0.34 keV due to C.

## 3. Experimental Section

### 3.1. Sample preparation

Bulk Si oxide samples were prepared by thermal oxidation of 6 inch Si wafers in an O_2_ environment at 900°C for 30 min. The oxidation time was chosen to grow a thin oxide layer, 6.5 nm thick, as measured by XPS [14, 20].

Porous silicon dioxide (PSiO_2_) samples were prepared using a homemade electrochemical cell, using H_2_O:HF:CH_3_CH(OH)CH_3_ (1:1:2) applying 1.5 A for 5 min to a 6 inch p^+^-type Czochralski Si wafer (ρ∼0.02 Ωcm). The electrochemical etching confers to the layer a “sponge-like” structure (see the insert of Figure 6). Finally, the samples underwent oxidation in a dry environment at 850 °C for 30 min.

The enzyme immobilization procedure cited in the literature [2] consisted of three steps: (1) silanization using 3-aminopropyltriethoxysilane (APTES); (2) linker molecule deposition and (3) enzyme coupling. To improve the immobilization protocol performance, we added a step (before the other three): oxide activation. It was carried out by immersing the samples in a solution (SSC) of NH_3_:H_2_O_2_:H_2_O (1:1:10) for 20 min. It increased the density of immobilized GOx as discussed previously. The second step of our protocol consisted in a treatment in APTES vapors. After silanization, samples were cured under vacuum to prevent APTES polymerization [14, 20]. The third step, linker molecule deposition, was carried out using glutaraldehyde (GA) 2.5% in phosphate buffer solution (PBS) 0.1M. Finally, in the forth step, samples were immersed in PBS solution containing GOx 2 mg/ml overnight at RT [13, 14]. GOx labelling with Au nanoparticles followed the NANOGOLD^®^ protocol as described elsewhere [20].

### 3.2. Enzymatic activity measurements

GOx activity was determined by measuring the amount of H_2_O_2_ formed, using a commercial spectrophotometric glucose assay kit, purchased from Megazyme^®^. GOx enzyme catalyzes the oxidation of glucose to D-gluconic acid producing hydrogen peroxide (see Introduction). In the presence of peroxidase (POD), hydrogen peroxide participates in a reaction involving *p*-hydroxy-benzoic acid and 4-aminoantipyrine (both provided in the kit) and a quinone imine dye complex is formed, which is measured at 510 nm. GOx was diluted in a homemade PBS buffer solution. To test the activity of the enzyme immobilized on the SiO_2_ samples, they were inserted in cuvettes containing the above mentioned peroxide solution.

### 3.3. Measurement equipments

X-ray Photoelectron Spectroscopy (XPS) analyses were carried out using a Kratos AXISHS spectrometer. In the present study the Mg Kα_1,2_ radiation of 1253.6eV was used at 10mA and 15keV and at pass energy of 40eV of the energy analyzer. The binding energy scale was referenced using the gold as internal reference, assuming Au4f_7/2_ peak at 84.0eV.

Scanning Electron Microscopy (SEM) measurements were performed using a LEO 1550 equipped with an Oxford 7426 EDX and with the INCA software program. The dried samples were cleaved to observe their cross section and placed in the microscope chamber under vacuum. No metal was deposited on the surface of the treated samples to minimize the number of treatments.

Absorbance measurements were carried out at 510 nm using a Varian Cary 50 spectrophotometer. GOx standard from Megazyme^®^ and GOx in a homemade dilution buffer (see before) were used to prepare the calibration curve. Absorbance was measured immediately after the reaction started and then every 10 min up to 4 hours. To monitor the immobilized GOx enzymatic activity, functionalized silicon samples (both bulk and porous SiO_2_) were placed in cuvettes containing both POD and D-glucose solutions. A cuvette containing only MilliQ water and a cuvette containing an unprocessed Si sample were used as references of GOx solution and of solid samples respectively, and measured using the same procedure described above. All measurements were carried out at room temperature. The absorbance values were normalized to the sample area.

### 3.4. Reagents

3-Aminopropyltriethoxysilane (APTES), glutaraldehyde solution (GA, 25% grade II), glucose oxidase (type X-S, *Aspergillus niger*, 179,000 U g-1 solid, Sigma), were purchased from Sigma Chemical Co. (St Louis, MI, USA). The other chemicals used were purchased from Carlo Erba Reagenti (Italy). Gold nanoparticles (1.4 nm in diameter) were purchased from Nanoprobes (Yaphank, NY, USA). Trialkylphosphine (TCEP) disulfide reducing gel, Handee^TM^ spin cup column, Handee^TM^ microcentrifuge tube and Elmann reagent were purchased from Pierce (Rockford, IL, USA). A commercial glucose assay kit was purchased from Megazyme^®^. Deionized, MilliQ water (18 MΩ resistivity) was used.

## 4. Conclusions

In conclusion, we have optimized a protocol to covalently immobilize the enzyme glucose oxidase on SiO_2_ and PSiO_2_ surfaces. We added an initial step to the procedure already reported in literature, which we call oxide activation, whose goal was to enhance the density of OH groups on the oxide surface. In fact, surface silanization (second step) occurs only on those groups. We determined, by XPS measurements, that a uniform organic layer was formed at the end of the immobilization procedure as demonstrated by a perfect shield of the Si° XPS by the organic layer. This result was not achieved when oxide activation was not carried out. The enzyme was correctly immobilized on the SiO_2_ surface, as indicated by both the C 1*s* signals at 286.3eV and 288.3eV assigned to RCH_2_*-NH-(CO)- and R-CH_2_-NH-(C*O)- groups, respectively. Au labelling was used as XPS internal reference and to provide a conclusive confirmation of GOx presence on the sample surface. From the enzymatic activity data we concluded that the immobilization procedure on SiO_2_ bulk supports did not denature or destabilize the enzyme and that the highest fraction of active enzyme was present if oxide activation was carried out. Moreover, enzymatic activity measurements demonstrated that our immobilization protocol is efficient and that the samples must be always stored in PBS at 4°C so that their activity can be retained for months.

Finally, a strong enhancement in the enzymatic activity was observed when PSiO_2_ surfaces were used, suggesting the pore permeation by GOx (indirect evidence). A conclusive proof was provided by SEM-EDX analysis that showed the C presence in the fully processed sample. This is a direct measurement of GOx presence within the pores.
